# Depleted Long Noncoding RNA GAS5 Relieves Intervertebral Disc Degeneration via microRNA-17-3p/Ang-2

**DOI:** 10.1155/2022/1792412

**Published:** 2022-03-15

**Authors:** Xiaojun Yu, Qikun Liu, Yinguang Wang, Yuan Bao, Yongqiao Jiang, Mengwei Li, Zhiwei Li, Baodong Wang, Lei Yu, Shanxi Wang, Ming Shao, Hao Kang

**Affiliations:** ^1^Department of Orthopedics, Tongji Hospital, Tongji Medical College, Huazhong University of Science and Technology, Wuhan 430030, China; ^2^Department of Orthopedics, The First Affiliated Hospital of Harbin Medical University, Harbin 150001, China; ^3^Department of Vascular Surgery, The First Hospital of Qiqihar (The Affiliated Qiqihar Hospital of Southern Medical University), Qiqihar 161000, China; ^4^Department of Orthopaedics, The Third Affiliated Hospital of Guangzhou Medical University, Guangzhou 510150, China

## Abstract

Intervertebral disc degeneration (IVDD) remains a clinical challenge and requires more effective therapeutic targets. Long noncoding RNAs (lncRNAs) have emerged as critical modulators of multiple biological processes, such as cell proliferation and extracellular matrix (ECM) remodeling. Accordingly, the current study sets out to explore the influence of the lncRNA growth arrest-specific 5 (GAS5) on IVDD and investigate the possible involvement of microRNA-17-3p (miR-17-3p)/Angiopoietin-2 (Ang-2) axis. Firstly, the expression patterns of GAS5, miR-17-3p, and Ang-2 were characterized by RNA quantification from the isolated human degenerative nucleus pulposus (NP) tissues. miR-17-3p was found to express at an abnormal low level while GAS5 and Ang-2 expressed at aberrant high level in the human degenerative NP tissues. Utilizing dual-luciferase reporter, RNA immunoprecipitation, and pull-down assays, GAS5 was found to competitively bound to miR-17-3p and further upregulate the expression of Ang-2, a target gene of miR-17-3p. Employing gain- and loss-of-function approaches, their expressions were altered in human degenerative nucleus pulposus cells (NPCs), followed by IL-1*β* treatment, in order to identify their roles in NP cell proliferation, apoptosis, and ECM metabolism. Silencing of GAS5 expression restrained the levels of cleaved caspase-3, cleaved caspase-7, cleaved caspase-9, MMP3, MMP13, ADAMTS4, and ADAMTS5 and increased collagen II and aggrecan levels. *In vitro* experiments also revealed that GAS5 depletion inhibited apoptosis and ECM degradation in HDNPCs, while elevating the proliferation through downregulation of Ang-2 by increasing miR-17-3p. Furthermore, *in vivo* data further validated that either GAS5 silencing or miR-17-3p reexpression alleviated IVDD degree with the help of IVDD mouse models. Altogether, our findings substantiated that downregulation of GAS5 reduced NPC apoptosis and promoted ECM remodeling, ultimately ameliorating the IVDD *via* miR-17-3p-dependent inhibition of Ang-2. We hope our discoveries offer a fresh molecular insight that can aid the development of novel therapies against IVDD.

## 1. Introduction

A multifactorial degenerative disease of the spinal cord, intervertebral disc degeneration (IVDD) represents a tremendous clinical challenge [1]. Pathologically, IVDD is characterized by the presence of proinflammatory factors, loss of active nucleus pulposus (NP) or annulus fibrosus (AF) cells, extracellular matrix (ECM) degradation, and alterations in disc cell phenotypes [2]. Risk factors responsible for the occurrence of IVDD include a variety of factors, such as genetic polymorphisms, age, smoking habit, and weight gain [3, 4]. Meanwhile, the cytokine inflammatory mediator interleukin-1 beta (IL-1*β*) is known to be produced as a result of pyroptosis, one of cell death mechanisms, after the activation of the inflammasome [5, 6]. In addition, existing evidence suggests that overproduction of IL-1*β* can facilitate inflammatory process and further precipitate nucleus pulposus cell (NPC) apoptosis and ECM degradation, ultimately leading to IVDD and lower back pain [7]. Although numerous studies have been conducted in a bid to illuminate potential therapeutic strategies, identification of more prognostic biomarkers or biological targets may contribute to the development of individualized therapeutics for this disease [8].

Over the last decade, long noncoding RNAs (lncRNAs), a group of noncoding RNAs longer than 200 nucleotides in length, have emerged as crucial regulators in a wide range of biological processes throughout the human body [9]. It is also noteworthy that several lncRNAs, such as TUG1 and HCG18, are known to confer regulatory roles on the functions of NPCs in IVDD, including processes such as cell differentiation, apoptosis, proliferation, and ECM deposition [10]. Meanwhile, growth arrest-specific transcript 5 (GAS5), located at chromosome 1q25, has been acknowledged to function as a tumor suppressor in numerous cancers [11]. Moreover, GAS5 has also been implicated as a pathogenic lncRNA in inflammatory conditions, including osteoarthritis [12, 13]. A recent study further came across the ability of GAS5 to potentiate the apoptosis of NPCs *via* binding to miR-26-5p, such that GAS5 silencing was indicated as an effective therapeutic strategy for treating IVDD [14]. Similarly, GAS5 has been reported to interact with other miRNAs, including miR-18a-5p [15] and miR-17-5p [16], both of which belong to the miR-17-92a cluster. Furthermore, miRNAs have garnered considerable attention in regard to IVDD due to their extensive regulatory influences on NPC functions, such as ECM remodeling, proliferation, and apoptosis [17]. More notably, miR-17-3p is capable of suppressing NPC apoptosis and stimulating ECM formation, which underscores its diagnostic significance in IVDD [18]. Accordingly, we speculated whether GAS5 could function to regulate miR-17-3p to influence the fate of NPCs. Interestingly, online prediction results from the RNA22 database indicated the presence of miR-17-3p binding sites in the 3′untranslated region (3′UTR) of Angiopoietin-2 (Ang-2), which is known to possess the ability to suppress NPC adhesion and proliferation, as well as to promote NPC apoptosis [19]. Additionally, there is a plethora of evidence that suggests that Ang-2, also known as ANGPT2, can induce the release of IL-1*β* to inhibit IL-1*β*-evoked apoptosis and ECM degradation in NPCs [20–22]. In lieu of the aforementioned evidence, we further conjectured whether the GAS5/miR-17-3p/Ang-2/IL-1*β* axis may be associated with the initiation and progression of IVDD by affecting the biological functions of NPCs. Thereafter, we obtained clinical samples from patients with IVDD to characterize the expression patterns of the GAS5/miR-17-3p/Ang-2/IL-1*β* axis and conducted a series of *in vivo* and *in vitro* assays to elucidate their role in modulating the functions of NPCs and the progression of IVDD. With the patients underwent surgical operation owing to thoracolumbar fracture or scoliosis as control [23], this study characterized the differential expression of GAS5 in the NP tissues of IVDD patients before experiments.

## 2. Materials and Methods

### 2.1. Ethical Approval and Consent to Participate

All experimentation procedures in the current study were approved by the Ethics Committee of Tongji Hospital, Tongji Medical College, Huazhong University of Science and Technology, with written informed consents obtained from all participants prior to specimen collection. All experiment procedures involving animals were conducted under specific pathogen-free conditions at the Tongji Animal Center (Wuhan, China) in compliance with the International Guiding Principles for Animal Research and the regulations and guidelines of Animal Ethics Committee of Huazhong University of Science and Technology.

### 2.2. Study Subjects

A total of 65 human nucleus pulposus (NP) samples were collected from IVDD patients treated by discectomy (comprising of 37 males and 28 females, with a calculated mean age of 48.42 ± 8.96 years) and thoracolumbar fracture or scoliosis (comprising of 24 males and 25 females, with a calculated mean age of 51.45 ± 9.16 years). The clinicopathological characteristics of all patients are shown in Supplementary Table 1. The IVDD scores of the obtained NP samples were ranged between Pfirrmann grades II and IV.

### 2.3. Cell Treatment

Human degenerative NPCs (HDNPCs) were isolated from the collected human NP tissue samples by 30 min of treatment with 0.25% pronase (Sigma-Aldrich, St Louis, MO, USA) and then 4 h of detachment with 0.2% type II collagen (Invitrogen, Carlsbad, CA, USA). Subsequently, the harvested NPCs were cultured in 5% CO2 at 37°C after resuspension in Dulbecco's modified Eagle's medium (DMEM, Gibco, Grand Island, NY, USA) added with 10% fetal bovine serum (FBS, Invitrogen), 1% streptomycin (Sigma-Aldrich), 2 mM glutamine, and 50 *μ*g/mL vitamin C (Sigma-Aldrich). Afterwards, the subcultured cells at the logarithmic phase of growth were seeded in 6-well plates (at a density of 1 × 10^5^ cells/mL), followed by another 24 h of incubation.

Upon reaching about 70% confluence, the cells were transfected with the following plasmids using Lipofectamine 2000 reagent (Invitrogen): siRNAs against GAS5 or Ang-2 (si-GAS5 and si-Ang-2), si-negative control (NC) (si-NC), overexpression plasmid of GAS5 or Ang-2 (oe-GAS5 or oe-Ang-2), oe-NC, miR-17-3p mimic, miR-17-3p inhibitor, NC mimic, and NC inhibitor. After 48 h of transfection, IL-1*β* (10 ng/mL) was added for another 24 h incubation for the cells treated with si-NC, si-GAS5, si-Ang-2, NC mimic, or miR-17-3p mimic.

### 2.4. Mouse IVDD Model Establishment and Animal Grouping

A total of 64 male C57BL/6J mice (age: 5-6 months) were procured from the Animal Experimental Center of Hubei Center for Animal Disease Control and Prevention (Wuhan, China). Subsequently, 56 mice were used to establish IVDD models, while the remaining 8 mice were adopted as the control group. Following anesthesia induction with 3% pentobarbital sodium (P3761; Sigma-Aldrich), puncture surgeries were performed at the Co6/7, Co8/9, and the Co10/11 levels of intervertebral discs (IVDs) in the mice using 33-gauge needles under fluoroscopic guidance, followed by standard postoperative procedures [24]. The Co7/8 and Co9/10 levels were regarded as the intervening controls.

One week after surgery, the mice were intraperitoneally anesthetized with pentobarbital sodium, and those presenting with severe degenerated or nondegenerated IVDs under magnetic resonance imaging (MRI) were excluded from the experiment. The IVDs conforming to Pfirrmann grades II and III were further recorded and labeled [25]. The MRI signal intensity was graded according to the modified Thompson classification method, ranging from I to IV (I, normal; II, MRI signal intensity decreased slightly and significantly narrowed in the strong signal region; III, MRI signal intensity decreased moderately; and IV, MRI signal intensity decreased severely) [26].

Afterwards, plasmids of sh-NC, sh-GAS5, oe-NC, oe-GAS5, miR-17-3p mimic, and miR-17-3p inhibitor were packaged into lentivirus vectors (pSIH1-H1-copGFP and pLV-EGFP-N) by GenePharma Co., Ltd. (Shanghai, China) to generate lentiviruses including pSIH1-H1-copGFP-sh-NC, pSIH1-H1-copGFP-sh-GAS5, pLV-EGFP-NC, pLV-EGFP-GAS5, pSIH1-H1-copGFP-miR-17-3p inhibitor, and pLV-EGFP-miR-17-3p, respectively. Simultaneously, human embryonic kidney- (HEK-) 293T cells (Shanghai Cell Bank of Chinese Academy of Sciences, Shanghai, China) were cultured in 10% FBS-contained Roswell Park Memorial Institute- (RPMI-) 1640 complete medium for lentivirus packaging. Subsequently, the lentivirus (1 × 10^9^ pfu/100 *μ*L) was injected into the punctured IVDs of mice using 33-gauge needles. Duplicate injections were performed 4 weeks later, and the subsequent experiments were conducted after another 4 weeks. NP tissues of mice were extracted for subsequent experiments.

### 2.5. Immunohistochemical (IHC) Staining

Paraffin-embedded human NP tissue slices (4 *μ*m thick) were dewaxed in the conventional manner and subsequently subjected to antigen retrieval under both high temperature and high pressure with a citric acid-salt solution. After successive incubation with avidin and d-biotin, the endogenous peroxidase activity of the slices was inactivated with the addition of H_2_O_2_ for 15 min. Following blocking with 10% goat serum, the slices were probed at 4°C overnight using diluted rabbit monoclonal antibody against Ang-2 (ab8452, 1 : 200, Abcam, Cambridge, UK) and then probed at ambient temperature for 10 min with the biotin-labeled goat anti-rabbit IgG secondary antibody (ab6721, 1 : 1,000, Abcam). Subsequently, after incubation with streptomycin avidin-peroxidase solution, the slices were stained with freshly prepared diaminobenzidine (DAB) (ZSGB-BIO, Beijing, China), followed by hematoxylin counter-staining. Finally, after gradient alcohol dehydration, xylene clearing, and neutral balsam sealing, the slices were submitted to microscopical observation.

### 2.6. Dual-Luciferase Reporter Assay

The RNA22 online database (https://cm.jefferson.edu/rna22/Interactive/) was adopted in order to predict the binding relation between GAS5 3′UTR and miR-17-3p, as well as miR-17-3p and Ang-2 3′UTR, and the results of which were further validated with a dual-luciferase reporter assay. The sequences harboring the binding sites or the mutant form (MUT) were cloned into the pUC57 vector and then subcloned into the psiCHECK-2 vector. Following amplification, all plasmids were extracted using plasmid mini kits from Omega (D1100-50T, Solarbio Science & Technology Co., Ltd., Beijing, China). The HEK-293T cells seeded in 6-well plates (density of 2 × 10^5^ cells/well) were submitted to transfection with the luciferase reporter plasmids of GAS5-WT, GAS5-MUT, Ang-2-WT, and Ang-2-MUT, respectively, with miR-17-3p mimics. After a period of 48 h incubation, Genecopoeia's Dual-Luciferase Assay kits (D0010, Solarbio) were adopted for measuring the luciferase activity, while a Glomax 20/20 luminometer fluorescence detector (Promega Corp., Madison, WI, USA) was utilized for measuring the luminance values. The experiment was repeated in triplicate to obtain the mean value.

### 2.7. RNA-Binding Protein Immunoprecipitation (RIP) Assay

RIP kits (Millipore Inc., Bedford, MA, USA) were adopted in order to identity the binding affinity between GAS5 and Ang-2. Briefly, the HDNPCs were lysed on ice with a 100 *μ*L radioimmunoprecipitation assay (RIPA) lysis buffer (P0013B, Beyotime Biotechnology Co., Shanghai, China) for 5 min, which were subsequently subjected to 10 min of centrifugation (14,000 rpm at 4°C). Afterwards, the obtained cell lysates were incubated with the rabbit polyclonal antibody against IgG (ab202985, 1 : 1,000) or against Argonaute 2 (Ago2) (ab32381, 1 : 10,000) from Abcam Inc. for coprecipitation. Subsequently, 50 *μ*L of magnetic beads mixed with 100 *μ*L of RIP Wash Buffer were incubated with 5 *μ*g of antibody. The antibody-coated magnetic beads were resuspended with 900 *μ*L RIP Wash Buffer and then incubated overnight with 100 *μ*L of cell lysate at 4°C. The following day, after purification in proteinase K buffer, total RNA content was separated from the collected magnetic beads, followed by reverse transcription quantitative polymerase chain reaction (RT-qPCR) analyses.

### 2.8. RNA-Fluorescence In Situ Hybridization (FISH)

FISH kits (Roche, Basel, Switzerland) were further adopted to identify the subcellular localization and expression patterns of GAS5. Prior to hybridization, the NP tissues were digested with 0.8% pepsase at 37°C, hydrated, and permeated for 30 min. After fixation with neutral formaldehyde and incubation with hybridization solution containing digoxin-labeled GAS5 probes (Sigma-Aldrich), the nuclei in the NP tissues were subjected to 10 min of staining at ambient temperature with 4′,6-diamidino-2-phenylindole (DAPI, Sigma-Aldrich). Afterwards, microscopical observation was performed using a confocal laser scanning microscope (FV1000, Olympus Optical Co., Ltd., Tokyo, Japan). Blue (DAPI) and green (GAS5) fluorescence signals were obtained with the channels, and the fluorescence intensity was calculated by normalization to that in the control group (set as 1).

### 2.9. RNA Pull-Down Assay

The HDNPCs were transfected with 50 nM biotinylated GAS5-WT (Bio-GAS5-WT) and 50 nM biotinylated GAS5-MUT (Bio-GAS5-MUT), respectively. After 48 h of transfection, the harvested cells were rinsed with PBS and incubated with specific lysis buffer (Ambion, Austin, TX, USA) for 10 min. Subsequently, 50 mL sample cell lysate was subpackaged, and the lysate was incubated with M-280 streptavidin magnetic beads (S3762, Sigma-Aldrich) precoated with RNase-free bovine serum albumin (BSA) and yeast tRNA (Sigma-Aldrich) at 4°C for 3 h. Following incubation, the aforementioned magnetic beads were washed twice with precooled lysis buffer, washed thrice with low salt buffer, and then washed once with high salt buffer. Antagonistic GAS5 probe was employed as the NC. After the RNA content was extracted with the help of the TRIzol reagent, RT-qPCR was carried out to analyze the expression patterns of miR-17-3p in the eluted samples.

### 2.10. RNA Isolation and Quantification

Total RNA content was extracted from the human and mouse NP tissues and cells by means of TRIzol kits (Invitrogen). The obtained total RNA (including miRNAs and mRNAs) was then reverse-transcribed into complementary DNA (cDNA) using a TaqMan™ MicroRNA Reverse Transcription Kit (4366596, Thermo Fisher Scientific, Waltham, MA, USA) and High-Capacity cDNA Reverse Transcription Kit (4368813, Thermo Fisher Scientific), respectively. Subsequently, RT-qPCR was carried out with the SYBR® Premix Ex Taq™ (Tli RNaseH Plus) reagent kit (RR820A, Takara Bio Inc., Otsu, Shiga, Japan) on an ABI7500 real-time quantitative PCR system (ABI Company, Oyster Bay, NY, USA). The employed primer sequences are presented in Supplementary Table 2. U6 was employed as the internal control for miRNA quantification, with glyceraldehyde-3-phosphate dehydrogenase (GAPDH) utilized for mRNA quantification. The fold changes were calculated by means of relative quantification (the 2^-*ΔΔ*Ct^ method) [27].

### 2.11. Western Blot Analysis

Total protein content was extracted from the human and mouse NP tissues and HDNPCs with the help of RIPA kits (R0010, Solarbio), and the protein concentration was determined using a bicinchoninic acid (BCA) kit (GBCBIO Technologies Inc., Guangdong, China). Next, a total of 40 *μ*g protein was subjected to sodium dodecyl sulfate-polyacrylamide gel electrophoresis, and the separated proteins were transferred onto a polyvinylidene fluoride membrane. Afterwards, the membrane was blocked with Tris-buffered saline Tween-20 (TBST) replenished with 5% BSA at ambient temperature and then probed overnight at 4°C with the following diluted rabbit polyclonal antibodies purchased from Abcam: Ang-2 (ab8452, 1 : 200), cleaved caspase-3 (ab2302, 1 : 200), cleaved caspase-7 (ab2323, 1 : 200), cleaved caspase-9 (ab2324, 1 : 200), metalloproteinase (MMP) 3 (MMP3) (ab53015, 1 : 1,000), MMP13 (ab39012, 1 : 3,000), a disintegrin and metalloproteinase with thrombospondin motifs (ADAMTS) 4 (ADAMTS4) (ab185722, 1 : 1,000), ADAMTS5 (ab41037, 1 : 250), collagen II (ab34712, 1 : 5,000), aggrecan (ab3773, 1 : 100), and GAPDH (ab9485, 1 : 2,500) which was adopted as the internal reference. The following day, the membrane was incubated with the secondary antibody, goat anti-rabbit IgG antibody (ab205718, 1 : 5,000, Abcam) at ambient temperature. Subsequently, an enhanced chemiluminescence (ECL) agent was employed to visualize the protein bands. The gray value was measured using the Image J software, and the relative ratio of the target band to the GAPDH band was calculated. The experiment was repeated in triplicate in each group to obtain the mean value.

### 2.12. CCK-8 Assay for Cell Viability

The adherent cells at the logarithmic phase of growth were detached with 0.25% trypsin and then suspended at a density of 5 × 10^4^ cells/mL. Subsequently, the single-cell suspension was seeded in a 96-well plate at a density of 100 *μ*L/well, followed by incubation at 37°C with 5% CO2 for 24 h, 48 h, and 72 h. Afterwards, 1 h prior to terminating the incubation, 10 *μ*L CCK-8 solution (Beyotime) was added to the individual wells. Following incubation, the optical density (OD) values were measured using an enzyme-labeling instrument (Becton, Dickinson and Company, NJ, USA) at a wavelength of 450 nm. Each group was set with 5 duplicates, and the mean value was recorded.

### 2.13. TUNEL Assay for Cell Apoptosis

TUNEL detection kits (Beyotime) were adopted for evaluating the apoptosis of both human NPCs and mouse NP tissues. Briefly, the treated mice were intraperitoneally euthanized with 3% pentobarbital sodium, and NP tissues were collected. Subsequently, the mouse NP tissues were paraffin-embedded, cut into slices, and dewaxed using the conventional method. Afterwards, the human NPCs were subjected to fixation with 0.1% Triton X-100 containing phosphate-buffered saline (PBS). Next, protease K (50 *μ*L; 20 *μ*g/mL; Sigma-Aldrich) was used to treat the mouse NP tissue slices and human NPCs, followed by antigen retrieval with a citric acid-salt solution. After 1 h of incubation with 50 *μ*L of TdT enzyme reaction solution in conditions void of light at 37°C, all samples were subjected to 50 *μ*L of peroxidase-labeled antidigoxigenin for 30 min. After reaction termination, 10 min DAB staining and hematoxylin counterstaining were performed. Later, the cells of TUNEL positive were counted in 5 randomly selected nonoverlapping views, with the apoptosis rate calculated as the percentage of the apoptotic cell number relative to the total cell number.

### 2.14. Histological Analysis

Following fixation with 4% paraformaldehyde and paraffin-embedding, the mouse NP tissues were sliced into 5 *μ*m thick sections and then subjected to Safranin O-Fast Green staining. Subsequently, the tissue slices were dewaxed and hydrated, stained with hematoxylin (Beyotime), differentiated with a hydrochloric-alcohol solution, and counterstained with eosin (Beyotime). Afterwards, the histological appearances of mouse NP tissues were observed and photographed using an optical microscope (DMI3000, Leica, Wetzlar, Germany), followed by assessment according to the scoring system reported by Masuda et al. [28].

### 2.15. Statistical Analysis

Measurement data were analyzed using the SPSS 21.0 statistical software (IBM Corp. Armonk, NY, USA). For statistical description, measurement data were summarized in a form of mean ± standard deviation. Measurement data between two groups were assessed using the independent sample *t* test, while that among multiple groups were analyzed by one-way analysis of variance (ANOVA) accompanied by Tukey's post hoc test. Repeated measures ANOVA with Bonferroni test was adopted to compare data among multiple groups at different time points. Pearson's correlation analysis was applied for analyzing the correlation between two indicators. A value of *p* < 0.05 was considered to be indicative of statistical significance.

## 3. Results

### 3.1. GAS5 Was Aberrantly Upregulated in the NP Tissues of Patients with IVDD

**Figure 1:**
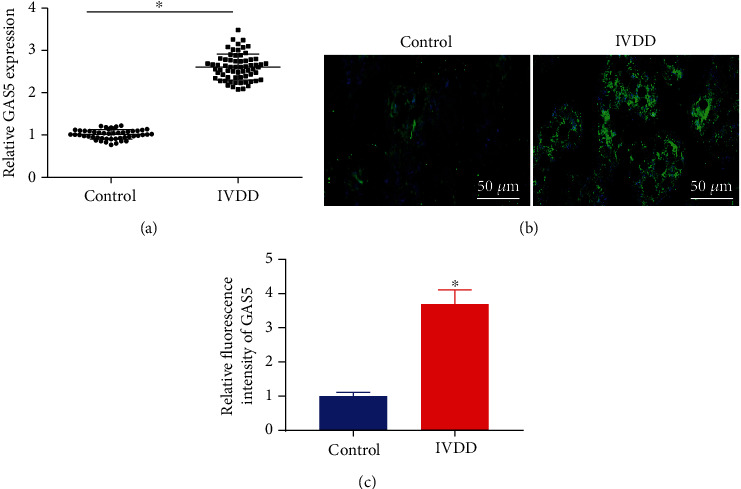
NP tissues of patients with IVDD exhibit high expression of GAS5. (a) Expression of GAS5 in NP tissues form 65 IVDD patients and control NP tissues from 49 patients with thoracolumbar fracture or scoliosis determined by RT-qPCR, which was normalized to GAPDH. (b) Expression of GAS5 in NP tissues form 65 IVDD patients and control NP tissues from 49 patients with thoracolumbar fracture or scoliosis detected by FISH (×200). (c) Proportion of GAS5+ cells in the NP tissues detected by RNA-FISH score, *n* = 3. ^∗^*p* < 0.05 vs. control NP tissues. Data (mean ± standard deviation) between two groups were compared using independent sample *t* test.

Firstly, the results of RT-qPCR illustrated that GAS5 was highly expressed in the NP tissues of patients with IVDD compared to that in the control NP tissues (Figure 1(a)). In addition, RNA-FISH was also carried out to determine GAS5 expression patterns in NPCs tissues, and as illustrated in Figures 1(b) and 1(c), the patients with IVDD presented with elevated GAS5 expression levels in NP tissues relative to control individuals (*p* < 0.05). Together, these findings unveiled GAS5 to be expressed at a high level in degenerative NP, highlighting its potential significance in IVDD.

### 3.2. GAS5 Inhibited Proliferation of NPCs and ECM Remodeling while Inducing Their Apoptosis

**Figure 2:**
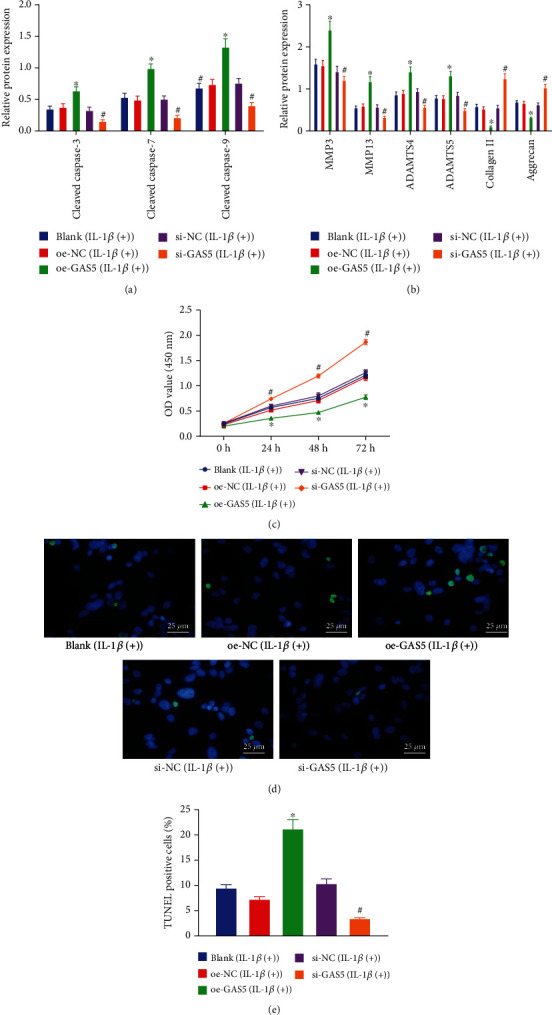
GAS5 stimulates NPC apoptosis and inhibits cell proliferation and ECM remodeling. (a, b) Western blot analysis of ECM metabolism-related and apoptosis-related proteins in NPCs, which was normalized to GAPDH. *n* = 3. (c) Viability of NPCs assessed by CCK-8 assay. *n* = 3. (d, e) TUNEL staining analysis of NPCs (×400). ^∗^*p* < 0.05 vs. HDNPCs treated with oe-NC and ^#^*p* < 0.05 vs. HDNPCs treated with si-NC. The cell experiment was repeated independently 3 times. Data (mean ± standard deviation) were assessed by one-way ANOVA, followed by Tukey's post hoc test. Data among groups at different time points were assessed with repeated measures ANOVA, followed by Bonferroni test. The experiment was repeated 3 times.

Given that the upregulation of GAS5 might serve as an important participant in IVDD as indicated by the aforementioned findings, we overexpressed GAS5 using an oe-GAS5 plasmid or knocked down GAS5 using siRNA in HDNPCs to evaluate its potential effects on IVDD. The levels of ECM metabolism-related proteins and apoptosis-related proteins (cleaved caspase-3, cleaved caspase-7, and cleaved caspase-9) were subsequently evaluated by means of Western blot analysis, and the results of which (Figures 2(a) and 2(b)) revealed that overexpression of GAS5 in NPCs brought about elevated protein levels of cleaved caspase-3, cleaved caspase-7, cleaved caspase-9, MMP3, MMP13, ADAMTS4, and ADAMTS5, while reducing those of collagen II and aggrecan (*p* < 0.05). Meanwhile, silencing of GAS5 in NPCs led to diminished levels of cleaved caspase-3, cleaved caspase-7, cleaved caspase-9, MMP3, MMP13, ADAMTS4, and ADAMTS5 proteins, in addition to elevated levels of collagen II and aggrecan proteins. Meanwhile, CCK-8 assay findings illustrated that cell viability was repressed as a result of GAS5 overexpression in NPCs, while increased viability was documented following loss of GAS5 (Figure 2(c)). Additionally, TUNEL staining was adopted to assess the effect of GAS5 on the apoptosis of HDNPCs, and as displayed in Figures 2(d) and 2(e), we observed that oe-GAS5 treatment led to increased apoptosis of NPCs, whereas GAS5-deficient cells presented with reduced apoptosis. Altogether, the abovementioned results indicated that GAS5 boosted cell apoptosis and inhibited cell proliferation and ECM remodeling in NPCs.

### 3.3. GAS5 Specifically Bound to miR-17-3p and Inhibited Its Expression

**Figure 3:**
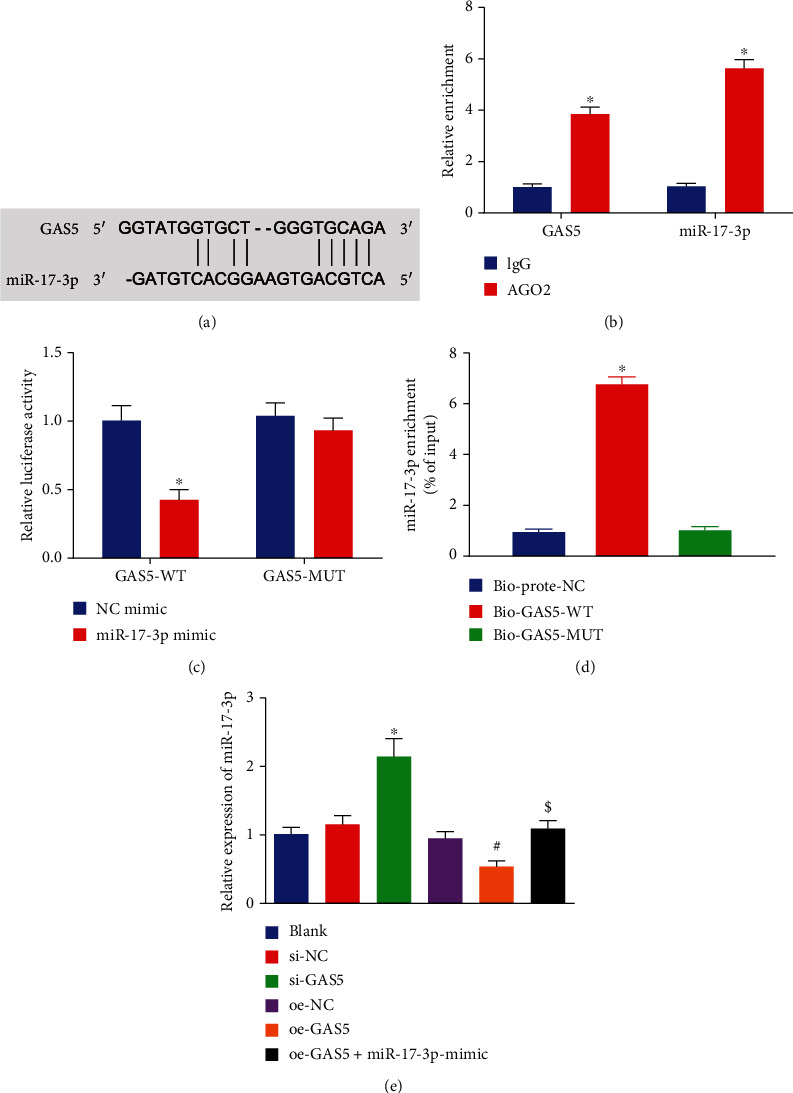
GAS5 specifically binds to miR-17-3p. (a) Binding sites of GAS5 and miR-17-3p predicted using the RNA22 database (https://cm.jefferson.edu/rna22/Interactive/). (b) Enrichment of miR-17-3p and GAS5 (^∗^*p* < 0.05 vs. HDNPCs incubated with rabbit polyclonal antibody to IgG) determined by RIP assay. (c) The luciferase activity of GAS-3′UTR reporter plasmids treated with miR-17-3p or control miRNA mimics determined by dual-luciferase reporter assay (^∗^*p* < 0.05 vs. HEK293T cells treated with NC mimic). (d) The enrichment of miR-17-3p (^∗^*p* < 0.05 vs. HDNPCs treated with biotinylated NC probes) detected by RNA pull-down assay. (e) Expression of miR-17-3p after the expression of GAS5 was altered (^∗^*p* < 0.05 vs. HDNPCs treated with si-NC). Data (mean ± standard deviation) in (b, c) were analyzed by *t* test, and those in (d, e) were assessed by one-way ANOVA, followed by Tukey's post hoc test. The cell experiment was repeated independently 3 times.

Existing evidence suggests that miR-17-3p possesses the ability to inhibit the apoptosis of NPCs [18]. Interestingly, the RNA22 online database predicted the existence of a binding site between miR-17-3p and GAS5 sequence (Figure 3(a)). To further verify the binding affinity of miR-17-3p to GAS5, we carried out a RIP assay to identify whether both of them could be coprecipitated in the Ago2-bound complex. Subsequent results demonstrated that compared with the anti-immunoglobulin G (IgG) control, NPCs incubated with anti-Ago2 presented with increased enrichment of miR-17-3p and GAS5 (Figure 3(b)). Meanwhile, the results of dual-luciferase reporter assay revealed that the luciferase activity of pisCHECK-2-GAS5 3′UTR was diminished following miR-17-3p mimic treatment. Upon miR-17-3p mimic treatment, insignificant differences were observed in the luciferase activity of pisCHECK-2-GAS5-MUT where the miR-17-3p potential binding sites were mutated (Figure 3(c)) (*p* > 0.05). Furthermore, a RNA pull-down assay was conducted to determine whether GAS5 could pull down miR-17-3p, and the obtained results (Figure 3(d)) further indicated that the cells manipulated with Bio-GAS5-WT presented with increased enrichment of miR-17-3p in relative to those manipulated with Bio-probe-NC (Figure 3(d)). Moreover, there were no differences in the enrichment of miR-17-3p between cells treated with Bio-probe-NC and Bio-GAS5-MUT (*p* > 0.05). Additionally, RT-qPCR results (Figure 3(e)) illustrated reduced expression levels of miR-17-3p as result of oe-GAS5 treatment, while elevated miR-17-3p levels were observed following GAS5 silencing. Lastly, we also found that the addition of miR-17-3p mimic could negate the reduced expressions of miR-17-3p elicited by GAS5 overexpression. Collectively, these findings demonstrated that GAS5 negatively regulated the expression of miR-17-3p by competitively binding to miR-17-3p in NPCs.

### 3.4. GAS5 Suppressed Proliferation and ECM Remodeling while Inducing Cell Apoptosis in NPCs via Downregulation of miR-17-3p

**Figure 4:**
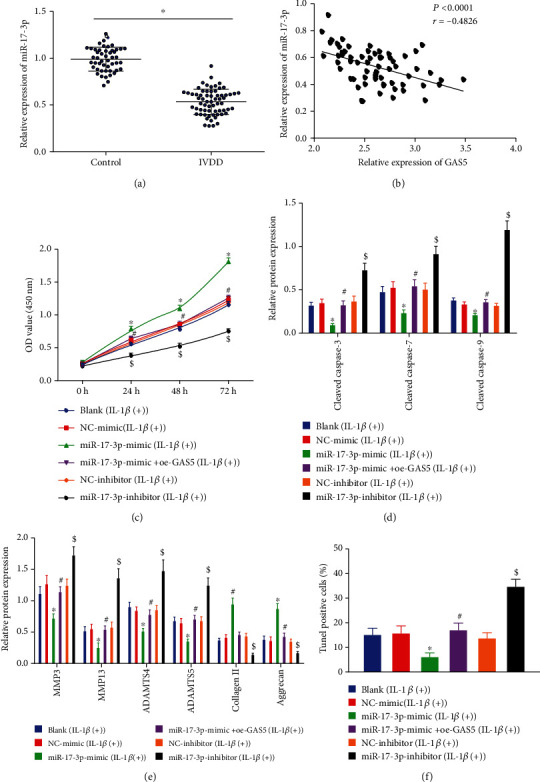
GAS5 augments NPC apoptosis while inhibiting proliferation and ECM remodeling by downregulating miR-17-3p. (a) Expression of miR-17-3p in 65 degenerative NP tissues and 49 NP tissues determined by RT-qPCR, which was normalized to U6. ^∗^*p* < 0.05 vs. the control NP tissues. (b) Correlation analysis on the expression in GAS5 and miR-17-3p in NP tissues of patients with IVDD using Pearson's correlation analysis, *n* = 65. (c) The viability of NPCs evaluated by CCK-8 assay. (d) Western blot analysis of apoptosis-related proteins, which was normalized to GAPDH. (e) Western blot analysis of ECM metabolism-related proteins, which was normalized to GAPDH. (f) Statistical results of TUNEL-positive NPCs. In (b, d–f), ^∗^*p* < 0.05 vs. HDNPCs treated with NC mimic, ^#^*p* < 0.05 vs. HDNPCs treated with miR-17-3p mimic, and ^$^*p* < 0.05 vs. HDNPCs treated with NC inhibitor. Data (mean ± standard deviation) in (a) were analyzed by independent sample *t* test, those in (b) were assessed by repeated measures ANOVA, followed by Bonferroni test, and those in (c–g) were assessed by one-way ANOVA, followed by Tukey's post hoc test. The experiment was repeated 3 times.

The aforementioned findings suggested that GAS5 might downregulate miR-17-3p in NPCs; thus, we accordingly focused on elucidating the potential effects of the GAS5/miR-17-3p axis on the functions of NPCs. As depicted in Figure 4(a), human degenerative NP tissues presented with decreased expression levels of miR-17-3p. Pearson's correlation analysis further revealed an inverse correlation between GAS5 expression and miR-17-3p expression in human degenerative NP tissues (Figure 4(b)). Meanwhile, the results of CCK-8 assay (Figure 4(c)) indicated that cell viability was elevated as a result miR-17-3p upregulation, whereas GAS5 overexpression could reverse the augmented cell viability stimulated by miR-17-3p upregulation (*p* < 0.05). Furthermore, as depicted in Figures 4(d) and 4(e), we observed that miR-17-3p mimic-treated cells exhibited decreased levels of caspase-3, cleaved caspase-7, cleaved caspase-9, MMP3, MMP13, ADAMTS4, and ADAMTS5, along with increased levels of collagen II and aggrecan, whereas all these trends were blocked when GAS5 was overexpressed simultaneously. In addition, manipulation with miR-17-3p inhibitor brought about elevated protein levels of cleaved caspase-3, cleaved caspase-7, cleaved caspase-9, MMP3, MMP13, ADAMTS4, and ADAMTS5 but reduced those of collagen II and aggrecan. Moreover, we adopted TUNEL staining to examine the potential effect of GAS5 on cell apoptosis *via* regulation of miR-17-3p, and the results of which (Figure 4(f)) illustrated cell apoptosis was reduced following miR-17-3p overexpression, while being boosted upon miR-17-3p downregulation. Consistently, GAS5 overexpression could reverse the inhibited cell apoptosis induced by miR-17-3p upregulation. Altogether, these findings confirmed that GAS5 inhibited NPC proliferation and accelerated NPC apoptosis and ECM degradation *via* downregulation of miR-17-3p.

### 3.5. GAS5 Upregulated Ang-2 Expression in HDNPCs by Negatively Mediating miR-17-3p

**Figure 5:**
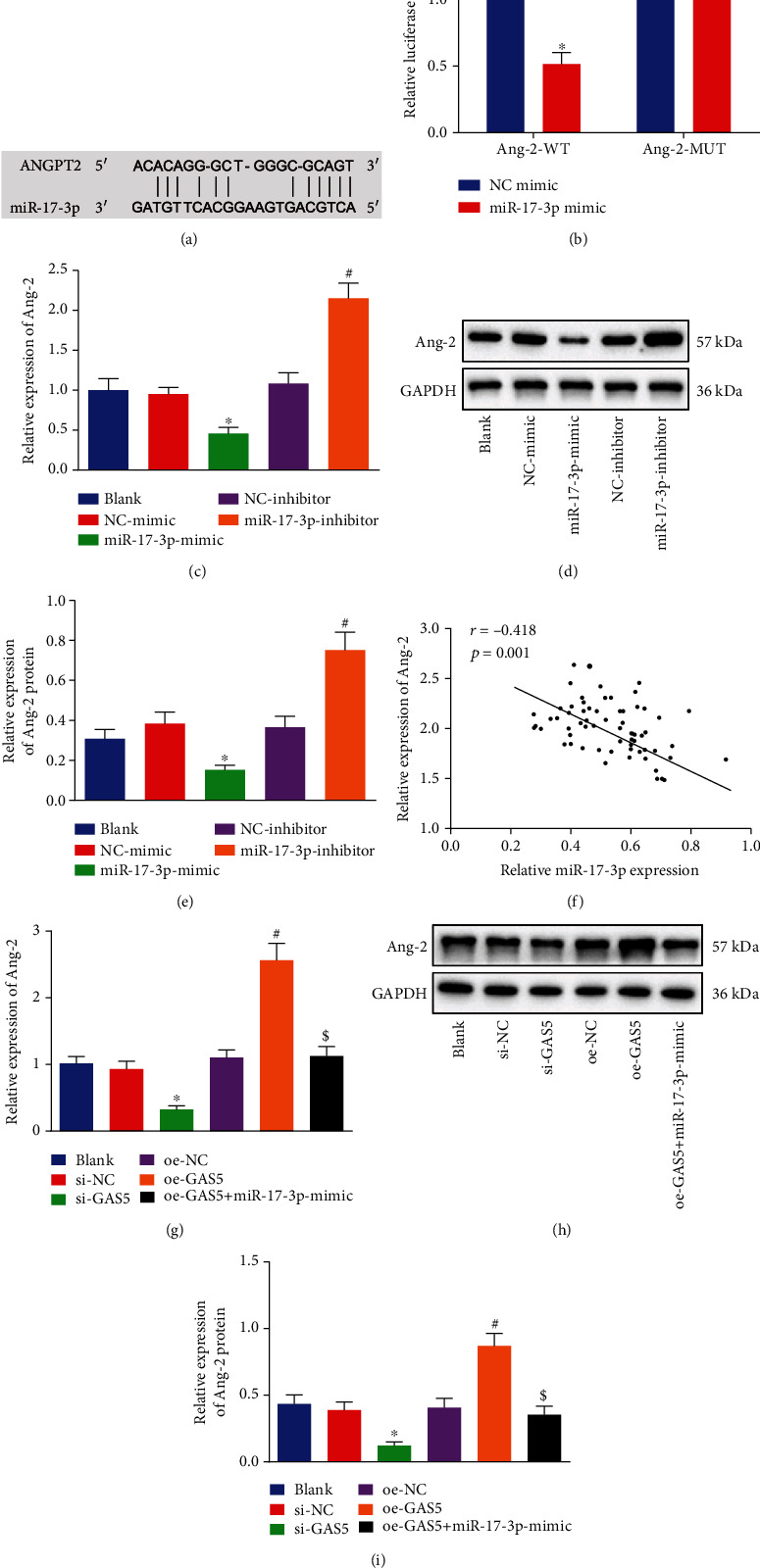
GAS5 upregulates Ang-2 expression in NPCs through downregulating miR-17-3p expression. (a) The binding sites between Ang-2 and miR-17-3p predicted using RNA22 website (https://cm.jefferson.edu/rna22/Interactive/). (b) The luciferase activity of Ang-2 reporter plasmids determined by dual-luciferase reporter assay (^∗^*p* < 0.05 vs. HEK 293T cells treated with NC mimic). (c) Expression of Ang-2 in NPCs after miR-17-3p was upregulated or downregulated and determined by RT-qPCR, which was normalized to GAPDH. (d, e) Western blot analysis of Ang-2 protein in NPCs after miR-17-3p was upregulated or downregulated, which was normalized to GAPDH. (f) Correlation analysis of miR-17-3p expression and Ang-2 expression in NP tissues. (g) Expression of Ang-2 in NPCs after GAS5 was upregulated or downregulated and detected by RT-qPCR, which was normalized to GAPDH. (h, i) Western blot analysis of Ang-2 protein in NPCs after GAS5 was upregulated or downregulated, which was normalized to GAPDH. ^∗^*p* < 0.05 vs. HDNPCs treated with si-NC, ^#^*p* < 0.05 vs. HDNPCs treated with oe-NC, and ^$^*p* < 0.05 vs. HDNPCs treated with oe-GAS5. Data (mean ± standard deviation) were analyzed by one-way ANOVA, followed by Tukey's post hoc test, while data in (b) were analyzed by unpaired *t* test. The cell experiment was repeated independently 3 times.

Based on the binding sites between miR-17-3p and Ang-2 3′UTR indicated by the RNA22 database (Figure 5(a)), we performed a luciferase assay to identify their binding relation. Subsequent findings illustrated a decline in the luciferase activity of pisCHECK-2-Ang-2 3′UTR caused by miR-17-3p mimic, while there were no obvious differences in that of pisCHECK-2-Ang-2 3′UTR-MUT in the miR-17-3p mimic-treated cells (Figure 5(b)). Moreover, as revealed in Figures 5(c)–5(e), mRNA and protein levels of Ang-2 were reduced following miR-17-3p overexpression, while opposing trends were observed when miR-17-3p was downregulated. Furthermore, we documented a converse correlation between the expression of miR-17-3p and that of Ang-2 (Figure 5(f)). Meanwhile, the results of RT-qPCR and Western blot analysis further demonstrated that Ang-2 expression levels were diminished as result of GAS5 downregulation, while Ang-2 was upregulated when GAS5 was upregulated. Besides, additional treatment of miR-17-3p was found to reverse the elevated Ang-2 levels elicited by GAS5 (Figures 5(g)–5(i)). Overall, these findings indicated that GAS5 might upregulate Ang-2 expression in HDNPCs by suppressing the expression of miR-17-3p.

### 3.6. siRNA-Mediated Silencing of GAS5 Accelerated NPC Proliferation and ECM Remodeling and Curbed Their Apoptosis by Downregulating miR-17-3p-Dependent Ang-2

**Figure 6:**
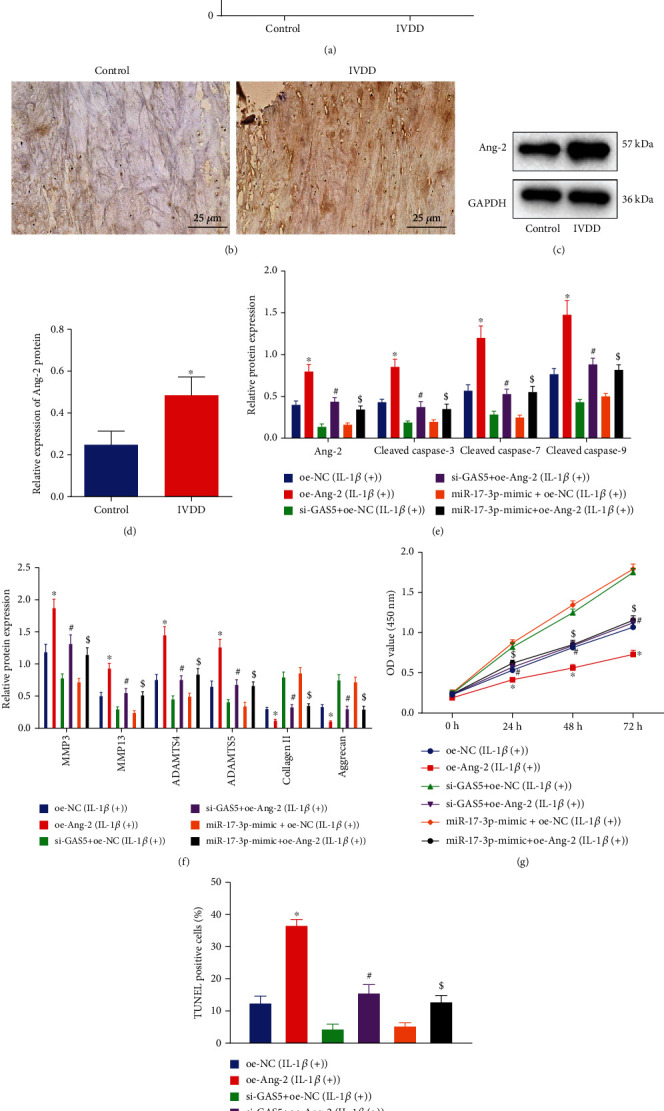
Silencing of GAS5 increases NPC proliferation and ECM remodeling but reduces NPC apoptosis via inhibiting Ang-2 and elevating miR-17-3p. (a) Expression of Ang-2 in 65 degenerative NP tissues and 49 control NP tissues by RT-qPCR, which was normalized to GAPDH. (b) Expression of Ang-2 in degenerative NP tissues and control NP tissues detected by IHC (×400). (c, d) Western blot analysis of Ang-2 protein in HDNPCs, which was normalized to GAPDH. (e) Western blot analysis of Ang-2 and apoptosis-related proteins in HDNPCs, which was normalized to GAPDH. (f) Western blot analysis of ECM metabolism-related proteins in HDNPCs, which was normalized to GAPDH. (g) The viability of NPCs assessed by CCK-8 assay. (h) Statistical results of TUNEL-positive NPCs. In (a, d) ^∗^*p* < 0.05 vs. the control NP tissues from patients with thoracolumbar fracture or scoliosis; in (e–h), ^∗^*p* < 0.05 vs. HDNPCs treated with oe-NC, ^#^*p* < 0.05 vs. HDNPCs treated with si-GAS5+oe-NC, and ^$^*p* < 0.05 vs. HDNPCs treated with miR-17-3p mimic+oe-NC. Data (mean ± standard deviation) in (a, d) were analyzed by independent sample *t* test, and those in (h) were assessed by repeated measures ANOVA, followed by Bonferroni test, and those in (f, g, i) were analyzed by one-way ANOVA, followed by Tukey's post hoc test. The cell experiment was repeated independently 3 times.

Ang-2 has been previously reported to facilitate ECM degradation in HDNPCs [22]. Accordingly, we focused on analyzing the involvement of Ang-2 in the proliferation and ECM remodeling of NPCs regulated by GAS5/miR-17-3p. The expression patterns of Ang-2 in NP tissue samples were determined by means of RT-qPCR, IHC, and Western blot analysis. As depicted in Figures 6(a)–6(d), human degenerative NP tissues presented with increased expression levels of Ang-2.

Furthermore, we observed that oe-Ang-2 treatment brought about elevated levels of Ang-2, cleaved caspase-3, cleaved caspase-7, cleaved caspase-9, MMP3, MMP13, ADAMTS4, and ADAMTS5 proteins, while reducing those of collagen II and aggrecan proteins (Figures 6(e) and 6(f)). Meanwhile, restoration of Ang-2 was found to counteract the changes in the levels of the aforementioned proteins caused by either loss of GAS5 or presence of miR-17-3p. Moreover, as shown in Figures 6(g) and 6(h), cell viability was reduced, while cell apoptosis was promoted following Ang-2 overexpression, whereas Ang-2 reexpression could negate the above effects of si-GAS5 or miR-17-3p mimic on viability and apoptosis. These findings provided evidence highlighting the involvement of the GAS5/miR-17-3p/Ang-2 axis in modulating the proliferation, apoptosis, and ECM remodeling of NPCs.

### 3.7. Silencing of GAS5/Ang-2 or Upregulated miR-17-3p Reversed Proapoptotic and Antiproliferative Effect of IL-1*β* on NPCs

**Figure 7:**
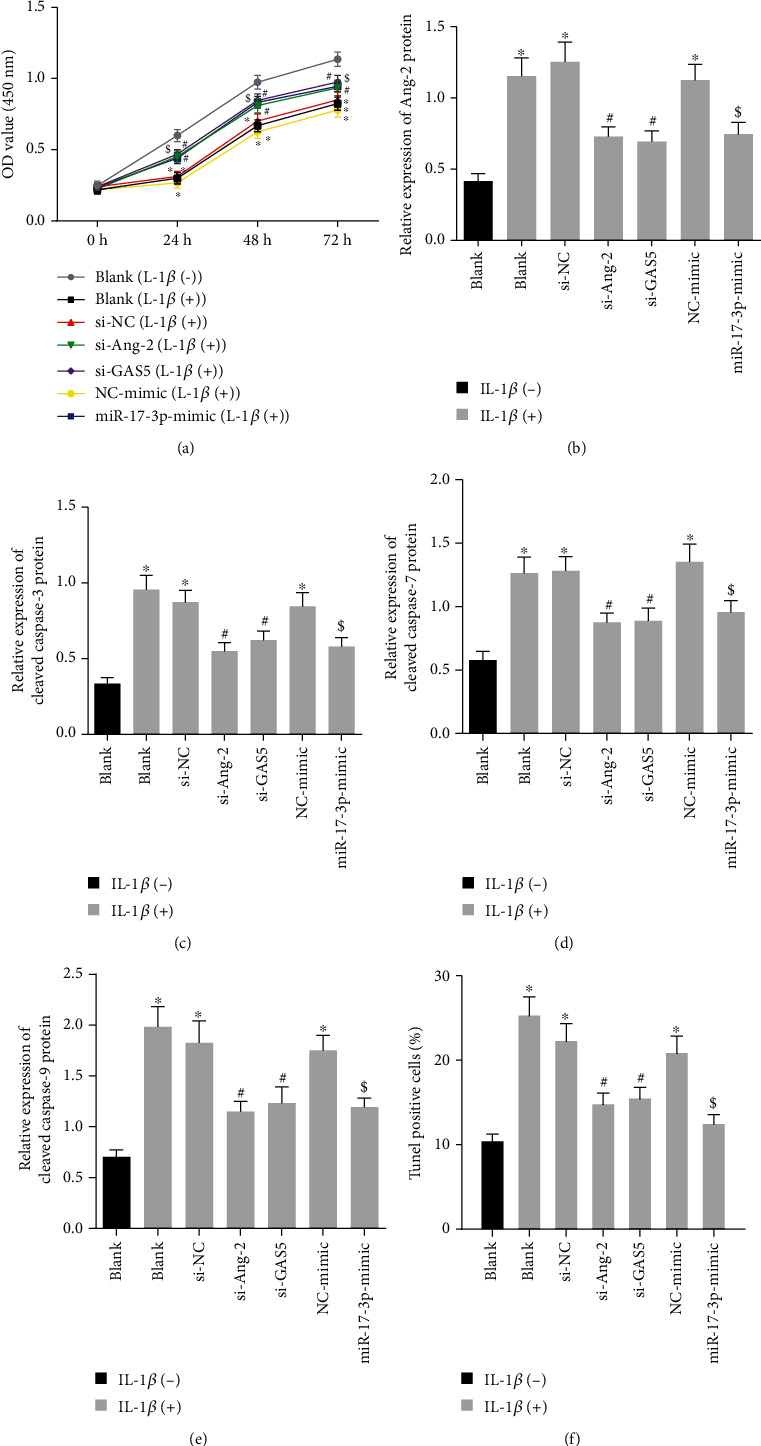
Downregulated GAS5/Ang-2 or elevated miR-17-3p arrests IL-1*β*-induced apoptosis and facilitates the proliferation of HDNPCs. (a) The viability of NPCs evaluated by CCK-8 assay. (b) Western blot analysis of Ang-2 protein in NPCs, which was normalized to GAPDH. (c) Western blot analysis of cleaved caspase-3 protein in NPCs, which was normalized to GAPDH. (d) Western blot analysis of cleaved caspase-7 protein in NPCs, which was normalized to GAPDH. (e) Western blot analysis of cleaved caspase-9 protein in NPCs, which was normalized to GAPDH. (f) Statistical results of TUNEL-positive NPCs. ^∗^*p* < 0.05 vs. HDNPCs without any treatment; ^#^*p* < 0.05 vs. HDNPCs treated with si-NC and 10 ng/mL IL-1*β*; ^$^*p* < 0.05 vs. HDNPCs treated with NC mimic and 10 ng/mL IL-1*β*. Data (mean ± standard deviation) were assessed by one-way ANOVA, followed by Tukey's post hoc test, while those in (a) were examined with repeated measures ANOVA, followed by Bonferroni test. The cell experiment was repeated independently 3 times.

Existing evidence suggests that Ang-2 possesses the ability to activate IL-1*β* [21], whereas overproduction of IL-1*β* is known to be associated with the loss of NPCs and ECM degradation [7]. Therefore, we intended to examine the effect of the GAS5/miR-17-3p/Ang-2 axis on IVDD process caused by IL-1*β*. Firstly, HDNPCs were incubated with 10 ng/mL of IL-1*β* to mimic the cellular context of IVDD, which revealed that cell viability was reduced after IL-1*β* treatment, while being rescued by silencing GAS5 or Ang-2 or by upregulation of miR-17-3p at the same time (Figure 7(a)). Moreover, as shown in Figures 7(b)–7(e), we observed that the protein levels of Ang-2, cleaved caspase-3, cleaved caspase-7, and cleaved caspase-9 were all increased following IL-1*β* treatment, whereas all of these changes were countered when GAS5 or Ang-2 was silenced or miR-17-3p was overexpressed (*p* < 0.05). Meanwhile, the results of TUNEL staining illustrated that cell apoptosis was enhanced after IL-1*β* treatment (*p* < 0.05) (Figure 7(f)), whereas si-GAS5 or si-Ang-2 and miR-17-3p mimic were found to reverse the elevated cell apoptosis induced by IL-1*β* (*p* < 0.05). Overall, these findings signified that elevation of miR-17-3p, as well as knockdown of GAS5 or Ang-2 could reverse the IL-1*β*-induced effects on NPC apoptosis and proliferation.

### 3.8. Silencing of GAS5/Ang-2 or Upregulated miR-17-3p Reversed the Impaired ECM Remodeling Induced by IL-1*β*

**Figure 8:**
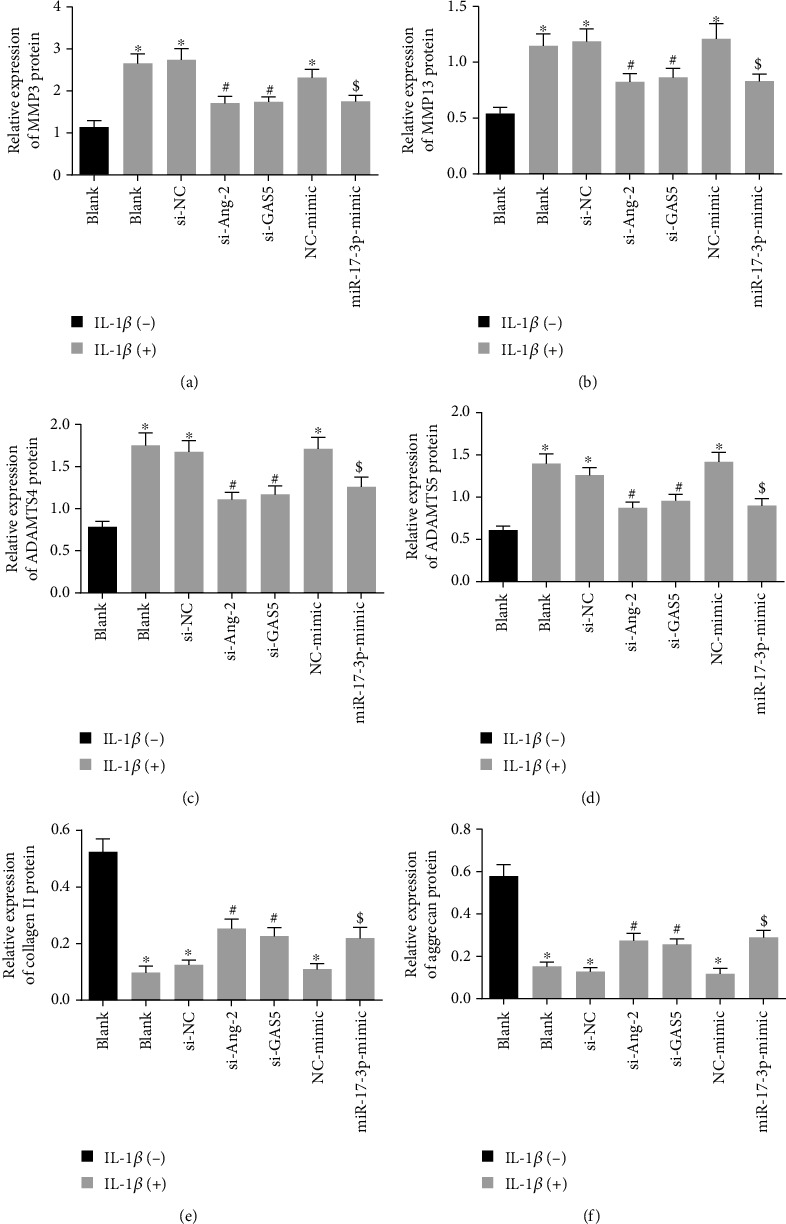
Downregulated GAS5/Ang-2 or elevated miR-17-3p rescues the inhibitory effect of IL-1*β* on ECM remodeling. (a) Protein levels of MMP3 in HDNPCs measured by Western blot analysis. (b) Protein levels of MMP13 in NPCs. (c) Protein levels of ADAMTS4 in HDNPCs measured by Western blot analysis. (d) Protein levels of E ADAMTS5 in HDNPCs measured by Western blot analysis. (e) Protein levels of collagen II in HDNPCs measured by Western blot analysis. (f) Protein levels of aggrecan in HDNPCs measured by Western blot analysis. ^∗^*p* < 0.05 vs. HDNPCs without any treatment; ^#^*p* < 0.05 vs. HDNPCs treated with si-NC and 10 ng/mL IL-1*β*; ^$^*p* < 0.05 vs. HDNPCs treated with NC mimic and 10 ng/mL IL-1*β*. Data (mean ± standard deviation) were assessed by one-way ANOVA, followed by Tukey's post hoc test. The experiment was repeated 3 times.

Furthermore, we assessed the levels of ECM metabolism-related proteins after IL-1*β* treatment and found that IL-1*β* treatment brought about elevated protein levels of MMP3, MMP13, ADAMTS4, and ADAMTS5 and reduced those of collagen II and aggrecan, whereas all these alterations could be reversed when Ang-2 was downregulated by si-GAS5, si-Ang-2, or miR-17-3p mimic (Figures 8(a)–8(f)). Taken together, these findings demonstrated that overexpression of miR-17-3p or silencing of GAS5 or Ang-2 reversed the ECM degradation elicited by IL-1*β*.

### 3.9. Silencing of GAS5 or Restoration of miR-17-3p Relieved IVDD In Vivo

**Figure 9:**
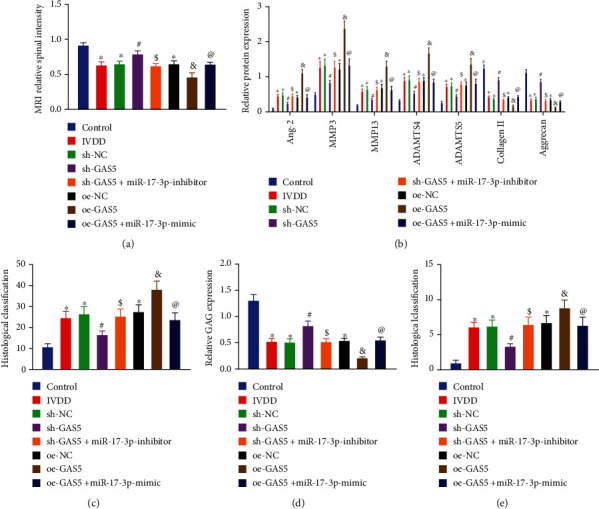
Silencing of GAS5 ameliorates IVDD in vivo by miR-17-3p-mediated inhibition of Ang-2. (a) Observation of the MRI signal intensity and IVDD degree by MRI scanning. (b) Western blot analysis of Ang-2 and ECM metabolism-related proteins in NP tissues extracted from IVDD mice, which was normalized to GAPDH. (c) Statistical results of TUNEL-positive cells in the NP tissues extracted from IVDD mice. (d) The expression of glucosaminoglycan (GAG) in mouse NP tissues detected by Safranin O-Fast Green staining. *n* = 8. (e) HE histological scores of mouse NP tissues by HE staining. *n* = 8. ^∗^*p* < 0.05 vs. mice without any injection; ^#^*p* < 0.05 vs. IVDD mice injected with harboring shRNA against shRNA NC; ^$^*p* < 0.05 vs. IVDD mice injected with lentivirus harboring shRNA against GAS5; ^$^*p* < 0.05 vs. IVDD mice injected with lentivirus harboring overexpression vector NC; ^@^*p* < 0.05 vs. IVDD mice injected with lentivirus harboring oe-GAS5. Data (mean ± standard deviation) were assessed by one-way ANOVA, followed by Tukey's post hoc test. *n* = 8.

Lastly, in order to assess the *in vivo* effects of the GAS5/miR-17-3p/Ang-2 axis on IVDD, lentiviruses harboring sh-GAS5, oe-GAS5, LV-miR-17-3p inhibitor, or LV-miR-17-3p were injected into the punctured IVDs of mice. MRI examination was subsequently performed for all mice (Figure 9(a)), which revealed that MRI signal intensity was decreased in the mice rendered with IVDD relative to the normal mice, which was indicative of aggravated IVDD degree. Meanwhile, we observed that MRI signal intensity in the IVDD mice was augmented by GAS5 knockdown, while being reduced by GAS5 overexpression. Moreover, miR-17-3p downregulation reduced the increase in MRI signal intensity induced by GAS5 knockdown in IVDD mice, while miR-17-3p overexpression was found to counteract the effects of GAS5 overexpression on MRI signal intensity and IVDD degree (*p* < 0.05).

Furthermore, NP tissues were extracted from mice and assessed using Western blot analysis, TUNEL, Safranin O-Fast Green, and HE staining. Subsequent results of Western blot analysis illustrated that the protein levels of Ang-2, MMP3, MMP13, ADAMTS4, and ADAMTS5 were all increased, while those of collagen II and aggrecan were diminished in the NP tissues extracted from IVDD mice as compared to those in the normal mice (*p* < 0.05; Figure 9(b)). Moreover, we found that the protein levels of Ang-2, MMP3, MMP13, ADAMTS4, and ADAMTS5 were all downregulated, while those of collagen II and aggrecan were augmented in the NP tissues from IVDD mice after GAS5 was silenced, whereas these trends could be reversed by additional downregulation of miR-17-3p. Meanwhile, when GAS5 was elevated, the protein levels of Ang-2, MMP3, MMP13, ADAMTS4, and ADAMTS5 were all elevated, and those of collagen II and aggrecan proteins were decreased, whereas these effects were countered by additional upregulation of miR-17-3p. Additionally, the results of staining experiments demonstrated that NP tissues extracted from IVDD mice presented with increased cell apoptosis, elevated ECM degradation, and raised histological scores compared to the control NP tissues. On the other hand, NP tissues obtained after GAS5 silencing in IVDD mice were found to exhibit reduced cell apoptosis, diminished ECM degradation, and decreased histological scores, whereas opposite changes were noted upon GAS5 upregulation. Importantly, those alternations induced by GAS5 loss-of-function could also be reversed by miR-17-3p knockdown, while those alternations induced by GAS5 gain-of-function were reversed by miR-17-3p reexpression (Figures 9(c)–9(e)). Altogether, the aforementioned findings highlighted the pivotal role of the GAS5/miR-17-3p/Ang-2 axis in relieving IVDD *in vivo*.

## 4. Discussion

Intervertebral disc degeneration (IVDD) is precipitated by the occurrence of a homeostatic imbalance along with upregulation of catabolic genes, resulting in dehydrated NP, decreased matrix components, and weakened AF [29]. Meanwhile, the hard-done work of our peers has highlighted the potential of targeting lncRNAs such as siRNAs or shRNAs against lncRNAs, antisense nucleotides, and small molecule inhibitors, as diagnostic and therapeutic markers in the management of IVDD owing to the diverse range of regulatory roles played by lncRNAs in human degenerative NP [30]. For instance, lncRNA taurine-upregulated gene 1 was previously demonstrated to be highly expressed in human degenerative NP tissues, whereas its downregulation was associated with a protective effect on human NPCs from tumor necrosis factor-alpha- (TNF-*α*-) induced apoptosis and senescence [31]. Similarly, another such lncRNA, namely, nuclear-enriched abundant transcript 1, is overexpressed in human degenerative NP tissues and further known to contribute to ECM degradation in HDNPCs, which reiterates the unfounded importance of lncRNAs in degenerative disorders [32]. In an effort to expand our knowledge on the same, the current study sets out to investigate the effects of GAS5 on NPC biological functions to elaborate its potential implications in the setting of IVDD. Ultimately, our findings revealed that GAS5 deficiency exerted weaker competitive binding ability to miR-17-3p to stimulate the downregulation of Ang-2, thus arresting IL-1*β*-induced NPC apoptosis and ECM degradation.

Firstly, our findings from a dual-luciferase reporter assay, in conjunction with online target prediction, revealed that GAS5 could function as a ceRNA of miR-17-3p. Inherently, miR-17-3p, a component of the miR-17-92 cluster, is known to be poorly expressed during the course of paraquat-induced dopaminergic neurodegeneration [33]. In addition, a prior study revealed that the miR-17-92 cluster was capable of mediating ECM proteins and subsequently influencing ECM modeling [34]. Meanwhile, sufficient levels of miR-17-3p are required for cardiac growth induced by exercise and further associated with a reduction in adverse cardiac remodeling to protect against myocardial ischemia-reperfusion injury [35]. It is also noteworthy that miR-17-3p has been shown to inhibit the apoptosis and ECM degradation of NPCs as well as to reduce the IVDD grade [18], which is largely in agreement with our findings. Additionally, our experiments provided data revealing that GAS5 could competitively bind to miR-17-3p. Interestingly, a number of have documented similar lncRNA-miRNA interactions, wherein lncRNAs serve as a ceRNA of miRNA in the pathogenesis of IVDD [36, 37].

Furthermore, our findings illustrated that GAS5 was highly expressed, while miR-17-3p was poorly expressed in NP tissues of patients with IVDD. In addition, we also noted that GAS5 silencing exerted a promotive effect on NP cell proliferation and ECM remodeling, while simultaneously conferring an inhibitory effect on NP cell apoptosis and ECM degradation, as evidenced by diminished levels of cleaved caspase-3, cleaved caspase-7, cleaved caspase-9, MMP3, MMP13, ADAMTS4, and ADAMTS5, and upregulated collagen II and aggrecan protein levels in HDNPCs. On the other hand, all the aforementioned trends could be reversed by silencing of miR-17-3p. Hence, it was concluded that GAS5 acted as a ceRNA of miR-17-3p thereby contributing to ECM degradation and NPC apoptosis. As primary enzymes, MMPs and ADAMTSs possess the ability to degrade collagens and aggrecan, while high levels of MMPs and ADAMTSs in NP tissues are associated with excessive ECM degradation during the occurrence and progression of IVDD [38]. Meanwhile, collagen II is one of the major macromolecules in IVDs, and its elevation is known to exert beneficial effects on degenerative conditions [39]. The proteoglycan aggrecan was previously shown to be responsible for maintaining swelling pressure to assist disc absorb shock and counter compressive loads [40]. In line with our findings, a prior study came across the ability of GAS5 to increase both the expressions of MMP2/3/9/13 and ADAMTS4 and further induce apoptosis in the setting of osteoarthritis pathogenesis [12], whereas its silencing is known to inactivate caspase-3 to resist cell apoptosis in neurons induced by oxygen-glucose deprivation, wherein GAS5 acts as a ceRNA of miR-137 [41]. Additionally, recent studies have shown that silencing of GAS5, which serves as a sponge of miR-26a-5p, contributes to augmenting the viability of degenerative NPCs and ECM synthesis [14]. Moreover, *in vivo* findings in our study further substantiated the potential therapeutic value of GAS5 loss-of-function in the form of suppressed ECM degradation and cell apoptosis and alleviated IVDD degree, while the opposing trends were noted as a result of miR-17-3p inhibition. In light of the aforementioned evidence, it would be plausible to suggest that GAS5 acts as a ceRNA of miR-17-3p, thereby contributing to ECM degradation and NPC apoptosis, while targeting GAS5 or elevating miR-17-3p may contribute to the development of new therapeutic strategies for IVDD.

Additional experimentation in our study revealed that GAS5 could upregulate the expression of the miR-17-3p target gene Ang-2 by competitively binding to miR-17-3p. This phenomenon was beautifully explained by the competing endogenous RNA (ceRNA) hypothesis, which stated that certain lncRNAs can function as a “sponge” of miRNAs and restrain miRNA-mRNA binding and thereby upregulate the expression of mRNAs [42]. Most importantly, we also uncovered that inhibition of Ang-2 expression could block the IL-1*β*-induced NPC apoptosis and ECM degradation in the presence of GAS5 silencing or miR-17-3p overexpression. Similarly, Wang et al. previously revealed that Ang-2 is profoundly expressed in degenerative samples, and this aberrant increase can impede NPC viability while stimulating their apoptosis [19]. Meanwhile, another study also documented that Ang-2 can increase IL-1*β* production [21]. Furthermore, existing evidence suggests that after IL-1*β* stimulation, NPCs present with reduced ECM remodeling in IVDD, aggrecan, and collagen II expression levels, in addition to upregulated levels of MMPs and ADAMTSs [43, 44]. Combining our *in vitro* findings with the previous data, it would be reasonable to suggest that GAS5 silencing may suppress IL-1*β*-induced NPC apoptosis and ECM degradation through the miR-17-3p/Ang-2 axis. Moreover, *in vivo* model experimentation in our study further corroborated that either silencing of GAS5 or restoration of miR-17-3p could indeed relieve the degradation of IVDs. Although several studies come across the regulatory effects of GAS5 and miR-17-5p on the expression of IL-1*β* [45, 46], very little is known about the GAS5/miR-17-3p/Ang-2/IL-1*β* axis in regard to the pathology of IVDD. Nevertheless, the newly identified GAS5/miR-17-3p/Ang-2 axis in our study sheds a new light on the potential mechanism implicated in the pathogenesis of IVDD, which might aid the development of appealing preventive targets for this disorder.

## 5. Conclusions

**Figure 10:**
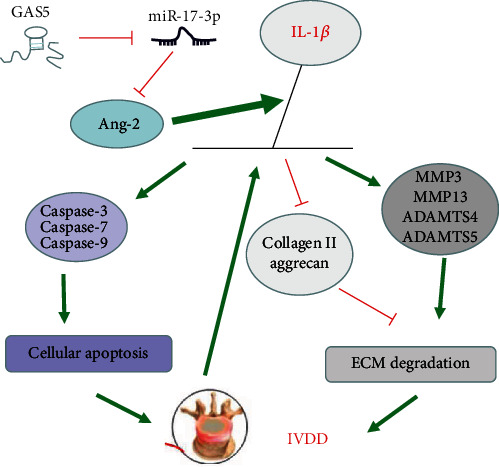
A mechanism map depicting the role of GAS5/miR-17-3p/Ang-2/IL-1*β* axis in IVDD. GAS5 impairs miR-17-3p-dependent Ang-2 and affects the expression of downstream IL-1*β*. Subsequently, the overproduction of IL-1*β* results in an elevation in the levels of ECM degradation-related proteins (MMP3, MMP13, ADAMTS4, and ADAMTS5) and apoptosis-related proteins (cleaved caspase-3, cleaved caspase-7, and cleaved caspase-9), along with dropped levels of ECM synthesis-related proteins (collagen II and aggrecan), thus leading to potentiated NP cell apoptosis and ECM degradation during IVDD.

Collectively, the key findings of the current study indicated that silencing of GAS5 inhibits IL-1*β*-induced NP cell apoptosis and ECM degradation through miR-17-3p-dependent repression of Ang-2 and ultimately relieves IVDD (Figure 10). We hope our findings may provide potential therapeutical basis for novel IVDD treatments in the form of targeted therapy aimed at GAS5 silencing or miR-17-3p overexpression.

## Data Availability

The datasets generated and/or analyzed during the current study are available from the corresponding author on reasonable request.

## Abbreviations

IVDD: Intervertebral disc degeneration
lncRNAs: Long noncoding RNAs
ECM: Extracellular matrix
GAS5: Growth arrest-specific 5
miR-17-3p: microRNA-17-3p
Ang-2: Angiopoietin-2
NP: Nucleus pulposus
NPCs: Nucleus pulposus cells
AF: Annulus fibrosus
3′UTR: 3′untranslated region
HDNPCs: Human degenerative NPCs
NC: Negative control
si-NC: si-negative control
IVDs: Intervertebral discs
MRI: Magnetic resonance imaging
HEK: Human embryonic kidney
IHC: Immunohistochemical
MUT: Mutant
RIP: RNA-binding protein immunoprecipitation
RIPA: Radioimmunoprecipitation assay
Ago2: Argonaute 2
RT-qPCR: Reverse transcription quantitative polymerase chain reaction
FISH: Fluorescence in situ hybridization
Bio-GAS5-WT: Biotinylated GAS5-WT
Bio-GAS5-MUT: Biotinylated GAS5-MUT
BSA: Bovine serum albumin
cDNA: Complementary DNA
GAPDH: Glyceraldehyde-3-phosphate dehydrogenase
BCA: Bicinchoninic acid
TBST: Tris-buffered saline Tween-20
ECL: Enhanced chemiluminescence
OD: Optical density
PBS: Phosphate-buffered saline
ANOVA: Analysis of variance
IgG: Immunoglobulin G
TNF-*α*: Tumor necrosis factor-alpha.

## Supplementary Materials

Supplementary Materials Supplementary Table 1: the clinicopathological characteristics of patients with IVDD. Supplementary Table 2: primer sequences for RT-qPCR.
