# Supplementary material for: Depleted Long Noncoding RNA GAS5 Relieves Intervertebral Disc Degeneration via microRNA-17-3p/Ang-2
Source: Oxid Med Cell Longev. 2022 Mar 15;2022:1792412. doi: 10.1155/2022/1792412 (PMC8941580; doi:10.1155/2022/1792412)
Supplement: Supplementary Materials — Supplementary Table 1: the clinicopathological characteristics of patients with IVDD. Supplementary Table 2: primer sequences for RT-qPCR. [file 1792412.f1.docx]

**SUPPLEMENTARY TABLE 1: The clinicopathological characteristics of patients with IVDD**

| Grade | Case | Age (year) | Gender | | Course of disease | Intervertebral segments | | |
| --- | --- | --- | --- | --- | --- | --- | --- | --- |
|  |  |  | male | female | (month) | L3/4 | L4/5 | L5/S1 |
| Pfirrmann II | 11 | 49.73 ± 9.54 | 4 | 7 | 20.73 ± 3.04 | 2 | 5 | 4 |
| Pfirrmann III | 20 | 49.80 ± 9.43 | 13 | 7 | 24.70 ± 3.71 | 4 | 10 | 6 |
| Pfirrmann IV | 26 | 48.54 ± 8.03 | 16 | 10 | 29.46 ± 4.94 | 4 | 11 | 11 |
| Pfirrmann V | 8 | 42.75 ± 9.33 | 4 | 4 | 37.75 ± 4.86 | 1 | 5 | 2 |

**SUPPLEMENTARY TABLE 2: Primer sequences for RT-qPCR**

| Gene | Forward | Reverse |
| --- | --- | --- |
| Human-GAS5 | 5'-TGTGTCCCCAAGGAAGGATG-3' | 5'-TCCACACAGTGTAGTCAAGCC-3' |
| Mouse-GAS5 | 5'-CACGTGTTCCATCCTGGTCA-3' | 5'-GTCAAGGAAGCCCACCATCA-3' |
| Human-miR-17-3p | 5'-ATTACGGACTGCAGTGAAGGCAC-3' | 5'-ATCCAGTGCAGGGTCCGAGG-3' |
| Mouse-miR-17-3p | 5'-CTCAACTGGTGTCGTGGA-3' | 5'-ACTTGTAGCTCAACT-3' |
| Human-Ang-2 | 5'-CCCTACGTGTCCAATGCTGT-3' | 5'-CCGCTGTTTGGTTCAACAGG-3' |
| Mouse-Ang-2 | 5'-AGAATAAGCAAGTCTCGCTTCC-3' | 5'-TGAACCCTTTAGAGGCTCGGT-3' |
| Human-U6 | 5'-CATCTGTGGAACCCTCCATTC-3' | 5'-GCACGTATCGGACCACTGAG-3' |
| Mouse-U6 | 5'-GATAAGACCATTCGGGTGAAGTT-3' | 5'-CCTCCGTCAGCTTGTACTGG-3' |
| Human-GAPDH | 5'-AATGAATGGGCAGCCGTTA-3' | 5'-ATGGAATTTGCCATGGGTGG-3' |
| Mouse-GAPDH | 5'-CAGGAGAGTGTTTCCTCGT-3' | 5'-TTGAATTTGCCGTGAGTGGA-3' |

Note: RT-qPCR, reverse transcription quantitative polymerase chain reaction; GAS5, growth arrest-specific transcript 5; F, forward; R, reverse; miR-17-3p, microRNA-17-3p; Ang-2, Angiopoietin-2; GAPDH, glyceraldehyde-3-phosphate dehydrogenase.
